# Understanding stock market instability via graph auto-encoders

**DOI:** 10.1140/epjds/s13688-025-00523-3

**Published:** 2025-02-19

**Authors:** Dragos Gorduza, Stefan Zohren, Xiaowen Dong

**Affiliations:** Oxford-Man Institute of Quantitative Finance, University of Oxford, Walton Street, Oxford, UK

**Keywords:** Financial networks, Financial instability, Systemic risk, Graph machine learning, Graph auto-encoders, Auto-encoders

## Abstract

Understanding stock market instability is a key question in financial management as practitioners seek to forecast breakdowns in long-run asset co-movement patterns which expose portfolios to rapid and devastating collapses in value. These disruptions are linked to changes in the structure of market wide stock correlations which increase the risk of high volatility shocks. The structure of these co-movements can be described as a network where companies are represented by nodes while edges capture correlations between their price movements. Co-movement breakdowns then manifest as abrupt changes in the topological structure of this network. Measuring the scale of this change and learning a timely indicator of breakdowns is central in understanding both financial stability and volatility forecasting. We propose to use the edge reconstruction accuracy of a graph auto-encoder as an indicator for how homogeneous connections between assets are, which we use, based on the literature of financial network analysis, as a proxy to infer market volatility. We show, through our experiments on the Standard and Poor’s index over the 2015-2022 period, that the reconstruction errors from our model correlate with volatility spikes and can be used to improve out-of-sample autoregressive modeling of volatility. Our results demonstrate that market instability can be predicted by changes in the homogeneity in connections of the financial network which expands the understanding of instability in the stock market. We discuss the implications of this graph machine learning-based volatility estimation for policy targeted at ensuring financial market stability.

## Introduction

Financial market instability has long interested investors, as, during financial crises, previously uncorrelated companies collapse in value together, which exposes the market to a systemic level of risk that goes beyond the level of the company and requires a more global outlook [1]. Understanding this market-wide systemic risk driven by asset co-movement requires framing connections between firms as formed by evolving ‘complex adaptive networks’ [2]. This turns instability detection into a network analysis task. These networks are usually constructed by transforming and filtering correlation matrices of price returns [3]. They capture key structures of the correlation matrices, and, at the same time they avoid potentially noisy features derived from correlations directly [4]. Changes in topological features of the resulting financial graphs, such as shorter diameters [3], higher average clustering coefficient [3, 5], or higher average Ricci curvature [6, 7], are shown to be positively linked to measures of market instability such as higher volatility.

This suggests that higher market instability correlates with breakdowns to previously homogeneous patterns of network connection. Homogeneity is defined here as regular connection patterns across the entire network [8]. In financial networks, homogeneous patterns mean connections are more likely to exist between companies of the same industry sectors and with similar returns [9]. Homogeneity-disturbing shocks may arise from new information in the form of news announcements about financial events or assets, and they affects assets co-movement by modifying the way investors form opinions about how companies are related and how they will co-evolve [10–12]. From this perspective, market instability can be associated with a modified underlying mechanism through which inter-company connectivity structures appear. Increase in heterogeneity in financial networks indicate higher instability [9] and has been shown to granger-cause increases in the Chicago volatility index (VIX see Abbreviations list at the end of the article) [13]. In that situation, any shock could be transmitted across the entire market and not be limited to a single sector [14, 15].

It is worth noting that the studies mentioned above all adopt a “network-only” approach, where the measure of homogeneity only concerns the network topology. However, this may not fully capture the effect of node type or node features on the formation of links across the network. Indeed, once network measures are constructed, they do not make further use of any form of node features. This means existing studies implicitly assume that all information about company nodes is captured by their connectivity. This discounts information from the underlying price returns which traditional models like multivariate GARCH [16] or HEAVY [17] successfully use in forecasting volatility. We address the open question of how to measure market instability using financial networks together with company characteristics such as returns.

The graph machine learning literature has provided tools for the joint learning of network and node level features [18]. However, their applications to financial networks remain mostly limited to individual stock level return forecasting, with very few works looking more globally at market wide volatility [19]. Other graph machine learning based approaches often primarily focus on directly forecasting and there is a lack of use of unsupervised models which can learn representations of market states as well as being usable for forecasting [20]. We propose to use a graph auto-encoder (GAE) [21] to capture both connectivity and node feature information as part of an edge reconstruction task. We propose to use the signal generated by the performance of this reconstruction task as an indicator to forecast out-of-sample volatility.

Our results show that the signal generated by our method increases the performance of a volatility forecasting model by at least 4% across 3 different volatility forecasting models. Based on our results, the performance of the GAE-based indicator is a useful tool to improve volatility forecasting models. As we present in Sect. 5, in this work, we train a GAE on a edge-reconstruction task at time period t and use it to reconstruct the connections at the next time period ($t + 1$). We are interested in seeing how informative the out of sample reconstruction score of the GAE is for volatility forecasting. The GAE performance metric we use is the area under receiver operand curve, a performance measure used in evaluating the edge-reconstruction ability in the graph machine learning literature [21, 22]. This metric helps us define good GAE performance by measuring how well is able to reconstruct an unseen set of connections between firms using patterns learnt from prior market connectivity. We train the GAE on an in-sample semi-supervised task of reconstructing a subset of unobserved edges from timestep t, before we apply the trained model on reconstructing the edges of time $t + 1$. We find the average in sample area under receiver operand curve reconstruction quality is 0.874 across our sample. This suggests that the model is able to reconstruct the in-sample connectivity patterns well and is sufficiently trained making its’ out of sample performance meaningful. We analyse the predictive results for volatility forecasting of the out of sample GAE performance indicator in Sect. 3. Good GAE performance on reconstructing the unobserved network at the next time step would indicate a more stable and homogeneous market as homogeneity in edge connectivity helps reconstruction performance. However if the GAE performs poorly in reconstructing structures of financial graphs of future time steps, we argue this indicates a shift in the way connections are formed in the future network. A worse reconstruction performance would then be correlated with the presence of a non-homogeneous, unstable network (hence market) defined in literature [5, 8, 9]. We present and analyse pre and post-volatility spike networks in 6 and 4 to illustrate what a low and high volatility network looks like as well as to make the case that a GAE model trained on the patterns of the pre-spike (low volatility) network will see a marked decrease in performance when applied to reconstruct the edge patterns in the high-volatility network. Overall, our research investigates the ability of an unsupervised node-embedding model to provide warning signals for increases in market instability.

## Problem setting

We consider the financial market as a financial network represented by a graph $\mathcal{G}=\{\mathcal{E},\mathcal{V}\}$, with nodes representing firms and edges the similarities or correlations between firm prices. In addition, we consider a matrix **X** where each row represents company-specific features - in our case stock returns. We calculate the return volatility at time y ($\text{RV}_{t}^{t+\Delta t}$) that is used throughout the following sections as a proxy for instability. This is a measure of the variance of the returns of the average price returns *r* over Δ*t* periods (see Sect. 5.1 for details of how returns are calculated). Our goal is to develop a predictive measure of the out-of-sample instability - as represented by market volatility - of the financial market given $\mathcal{G}$ and **X**. We forecast out-of-sample volatility at the hourly frequency as it is a frequency of interest for industry practitioners [1, 5, 11].

In order to jointly account for information contained in the features of the company returns **X** and the structure of the network used in prior studies [5, 6], we utilise the GAE [21]. This model learns vector representations of nodes using both topological information in $\mathcal{G}$ and feature information in **X** (see Sect. 5.2 for detailed description of the GAE model). We train the GAE on a binary edge reconstruction task described in Sect. 5.2. A low reconstruction error on this task would indicate that the GAE is able to learn an accurate representation of the structure of the network and node features. As the GAE performs better in proportion to the homogeneity of connections in a network, we interpret good GAE performance in edge-reconstruction as indicative of a more homogeneous pattern of edge formation across the network.

Homogeneous connectivity patterns are usually observed in low volatility periods where companies in related sectors are often exposed to similar fluctuations and overall more connected to each other than to companies outside their industries [9]. On the other hand, more volatile markets are characterised by highly perturbed correlation structures leading to networks with connection structures which are highly dissimilar from their company-level features reflected in their returns. As such we seek to detect when such a high heterogeneity situation occurs as it can be used as a good metric for instability increases.

With this understanding, we frame our task as building a time-dependent indicator for market volatility by finding a good proxy for heterogeneity in the connection patterns of $\mathcal{G}$ between different time periods. Using the information encoding properties of a GAE on a binary edge reconstruction task, we calculate $\mathbf{S}_{t+1}$, which we define $\mathbf{S}_{t+1}$ as the generalisation ability at the next time period - i.e. ${t+1}$ of a GAE model trained on data from time *t* when applied to a network from time $t+1$ and use it as a proxy for market volatility. In order to validate the usefulness of $\mathbf{S}_{t+1}$ as an indicator we test it’s performance at forecasting volatility in an out-of-sample setting. To prevent leakage of information between $\mathbf{S}_{t+1}$ and the volatility at the same time period $\text{RV}_{t}^{t+1}$, we look at $\text{log}(\text{RV}_{t}^{t+2})$, the volatility at the following period for our out-of-sample assessment.

## Results

### Negative correlation between market volatility and GAE reconstruction overtime

**Figure 1:**
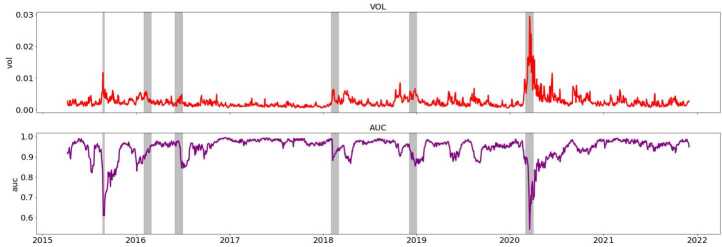
Time series of market volatility (top) and GAE reconstruction performance $\mathbf{S}_{t+1}$ (below)

Using the reconstruction accuracy of a GAE, we generate $\mathbf{S}_{t+1}$ which measures how much change in the homogeneity of the connection patterns of the financial networks from one time period *t* to the next one $t+1$. See Sect. 5.2 for an in depth description of the network processing and machine learning steps we follow to produce this reconstruction measure. Figure 1 presents the variation in market volatility (top) and in $\mathbf{S}_{t+1}$ overtime measure computed for a seven-year period from 2015 to 2022. The six grey bands in the Fig. 1 plot highlight periods of market instability over our sample. These are in chronological order: the August 2015 ‘flash crash’, the market reactions to the announcement (February 2016) and result (June 2016) of the Brexit referendum, the high volatility periods of the beginning and end of 2018 and lastly the 2020 pandemic stock market collapse.

We can see that the two time series comove strongly overtime as their peaks and troughs are seem to occur at similar times. They are also similar in their values overtime as the peaks in volatility are reflected in plunges of the same relative shape in the GAE reconstruction time series. $\mathbf{S}_{t+1}$ is high in periods of relative market stability where volatility is lower and sharply falls during high-volatility markets periods, usually associated with market-wide downturns. Figure 1 also shows the sharp troughs in $\mathbf{S}_{t+1}$ coinciding with market downturns depicted in grey. Highlighting these periods of market instability allows us to remark that the $\mathbf{S}_{t+1}$ seems to dip slightly before the band identifying a volatility upswing. This effect is noticeable especially for the bands identifying the 2016 Brexit announcement and the 2020 Covid-19 market upheaval.

**Figure 2:**
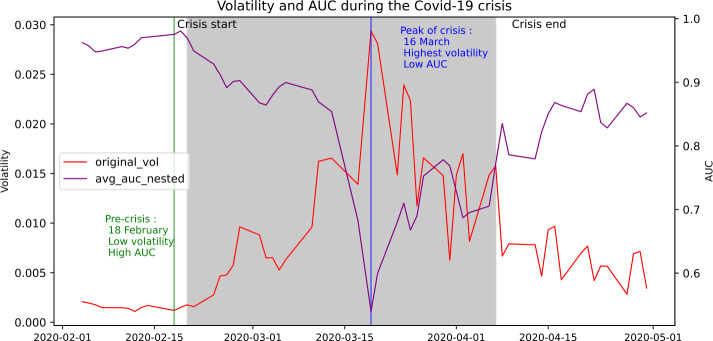
Case study on the co-movement of the volatility and reconstruction accuracy during the covid-19 stock market crash

Figure 2 highlights the strong co-movement between the GAE reconstruction accuracy in purple and the volatility in red during the 2020 Covid stockmarket contraction. This period saw an intense selloff by investors accross all asset classes associated with the arrival of Covid-19 in the us which resulted in an intense spike in volatility. The market network during this crisis displays similar behaviour as what can be observed in Fig. 6: a regular network with high segmentation between industries during the pre-crisis period up to February 18th indicated in green in our plot and a very tightly clustered network during the high volatility period indicated as a grey band. The peak of the volatility on March 16th indicated in blue coincides with the trough of the AUC indicator. Moreover, as suggested by the overall timeseries comparison in Fig. 1, the purple line begins to dip very quickly after the start of the crisis.

**Figure 3:**
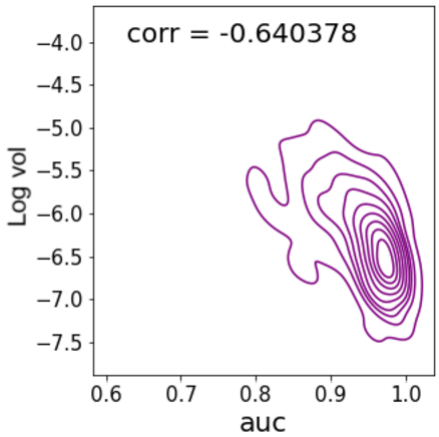
Kernel Density Estimation of $\mathbf{S}_{t+1}$ compared to volatility

We interpret the co-movement between these two time series based on the discussion above and on the literature that suggests the link between highly heterogeneous connection patterns and increases in volatility [9]. The synchronised variations between the two time series suggest that the GAE model is sensitive to the volatility in the market. A high value of $\mathbf{S}_{t+1}$ (purple) signifies that our GAE model’s reconstruction performance on the ${t+1}$ state of the network is high and the network is homogeneous. Conversely, a low value indicates a low reconstruction performance and a lower network homogeneity. This suggests that the volatility in the network disrupts the homogeneity of the network. This observation is reinforced by the simultaneity between troughs in $\mathbf{S}_{t+1}$ and market-wide crashes. It provides further evidence for our hypothesis of the GAE’s reconstruction performance acting as an indicator of market disruption. The slight dip ahead of certain crashes also suggests a potentially useful application of $\mathbf{S}_{t+1}$ in look-ahead forecasting of volatility. We further investigate this potential use of $\mathbf{S}_{t+1}$ in out of sample forecasting in the following sections. We interpret the behaviour of the AUC during the Covid-19 stockmarket crash as further indication of the strong negative relationship between market instability and the reconstruction ability of the model. The correlation between these two timeseries over this smaller period is −0.72, which is even higher than the overall long-run correlation coefficient displayed in Fig. 3, suggesting that the link between volatility and reconstruction ability is particularly strong during this crisis event. The AUC time series is below it’s long-run average of 0.84 for a majority of the crisis period suggesting it could serve as a good crisis indicator when it ‘dips’ below this value.

Figure 3 shows the kernel density estimation (KDE) and spearman’s rank correlation between log of the volatility and the $\mathbf{S}_{t+1}$. We can observe from Fig. 1 a −0.64 negative correlation between market volatility and our GAE based indicator. Furthermore, the KDE displays a strongly negative slope with increases in $\mathbf{S}_{t+1}$ being linked to decreases in volatility.

We interpret this negative correlation and the KDE as a sign of the mismatch between the ability of our model to reconstruct the network pattern in the market and the volatility of the market at time ${t+1}$. As the GAE struggles to learn a uniformly good latent node representation for a network at the same time period if the connections are not homogeneous, the $\mathbf{S}_{t+1}$ is strongly negatively correlated with the volatility [8]. The relationship between log volatility and the $\mathbf{S}_{t+1}$ described by the correlation and kernel density plot in Fig. 3 displays an inverse relation, with a spearman rank correlation of −0.64, between market volatility and the GAE reconstruction performance. This provides evidence in support of the expectation that the GAE produces a good measure for market instability as it encodes graph homogeneity, and drops in $\mathbf{S}_{t+1}$ are correlated with increased volatility.

### GAE reconstruction for out-of-sample market volatility forecasting

The correlation observed in the previous section motivates us to investigate the usefulness of the $\mathbf{S}_{t+1}$ in an out-of-sample setting. We use $\mathbf{S}_{t+1}$ as an explanatory variable to predict $\text{log}(\text{RV}_{t}^{t+2})$, the following period’s volatility. To this end, we fit multiple machine learning models as baselines following practice in the financial industry for volatility forecasting. The output variable of these models is $\text{log}(\text{RV}_{t}^{t+2})$ and the input variables include lagged averaged values of volatility of the previous period. To compare against the above baselines, we consider our variable of interest $\mathbf{S}_{t+1}$ as an additional variable (the detailed model is presented in Sect. 5.2).

Table 1 presents the results of the log-RV forecasting task at time period $t+2$ defined in the Methods section (see Eq. (6) and Eq. (8)). The *p*-value corresponds to a bootstrapped estimate of the significance of the difference between the $R^{2}$ of the prediction in a one-sided statistical significance test (see Sect. 5.4 for more detail), i.e., whether the $R^{2}$ of the machine learning forecasting method used with the added $\mathbf{S}_{t+1}$ is significantly larger than the $R^{2}$ without $\mathbf{S}_{t+1}$. We test three different machine learning forecasting strategies this way: a linear regression referred to as the linear model, a gradient boosted tree refered and a multi-layer perceptron referred to as the MLP model. The results show a statistically significant positive effect of the $\mathbf{S}_{t+1}$ on the forecasting of hourly volatility at time period $t+2$. The result aligns with the hypothesis of the problem setting and observations of the previous sections.

**Table 1:** Results of log-RV forecasting at the 1-hour frequency

Model	$R^{2}$ with $\mathbf{S}_{t+1}$	$R^{2}$ without $\mathbf{S}_{t+1}$	*p*-value of difference
Linear	0.500^∗^	0.452	0.03
Tree	0.510^∗^	0.450	0.00
MLP	0.565^∗^	0.521	0.001

Note: ^∗^indicate *p*-value < 0.05.

We also assess for the importance of node-level and edge-level information by carrying out an ablation study. This ablation study shows the performance of $\mathbf{S}_{t+1}$ as an explanatory variable for the volatility forecasting task under two settings, one where the information of the return matrix is absent and one where the information of the adjacency matrix is absent. We carry out the same experiments as in Table 1 above, while removing information from the network used to train the GAE and compute $\mathbf{S}_{t+1}$. First, we test for the importance of node level information by removing the node information in the market network by replacing the company return matrix **X** with an identity matrix. Then, we test for the importance of edge-level features by rewiring the patterns of edge connection of the market network.

Table 2 presents the result of the log-RV forecasting at time period $t + 2$ for the three machine learning models used in Table 1 in the ablation study where the node feature information was removed from the network. Table 2 shows that $\mathbf{S}_{t+1}$ without node feature information does not show statistically significantly higher $R^{2}$ scores at the 0.05 significance level compared to the linear and gradient boosted tree models. However, the table shows a statistically significant difference at the 0.05 level for the MLP. Table 3 presents the results of the ablation study where the edge information was removed from the network. It shows that the $\mathbf{S}_{t+1}$ generated by applying the GAE to the network with randomised edges performs better than the baseline linear model at the 0.05 significance level. It also outperforms the MLP model at the 0.05 confidence level. However, it does not show significantly higher results from the gradient boosted tree approach at the 0.05 significance level.

**Table 2:** Results of log-RV forecasting at the 1-hour frequency for network with ablated feature information

Models	$R^{2}$ with $S_{t+1}$	$R^{2}$ without $S_{t+1}$	p-value of difference
Linear	0.463	0.452	0.618
Tree	0.333	0.450	0.687
MLP	0.550^∗^	0.521	0.042

Note: ^∗^indicate *p*-value < 0.05.

**Table 3:** Results of log-RV forecasting at the 1-hour frequency for network with rewired edges

Models	$R^{2}$ with $S_{t+1}$	$R^{2}$ without $S_{t+1}$	p-value of difference
Linear	0.540^∗^	0.452	0.00
Tree	0.444	0.450	0.698
MLP	0.571^∗^	0.521	0.001

Note: ^∗^indicate *p*-value < 0.05.

The results from the ablation study show that the $\mathbf{S}_{t+1}$ reconstruction measure computed without using either edge or feature information functions poorly as an out-of-sample volatility estimator when added to the three machine learning models for volatility forecasting. This suggests that the information content present in the edges and the nodes is jointly important for the downstream out-of-sample performance of the $\mathbf{S}_{t+1}$ as a volatility prediction feature. However, the results for the edge-ablated model do show some improvement on average over the classical model. This suggests that the model is able to retain explanatory power better when deprived of edge-level information than when deprived of node level information and that edge-level information is less critical to the good performance of the out of sample forecasting.

## Discussion

Our research investigates the link between the encoding ability of an unsupervised node-embedding model and market instability. The results above support our hypothesis that the edge reconstruction accuracy of a GAE can serve as a good proxy to detect changes in the homogeneity of connectivity patterns across a financial network and thus serve as a good indicator for changes in market volatility. The GAE’s joint encoding of market returns and connectivity at time step $t+1$ is correlated with market instability at the same time step which we show is useful in forecasting out-of-sample volatility. Moreover, the results show that the reconstruction accuracy of the GAE can act as a good instability indicator as evidenced by its’ tendency to plunge before a crisis. As shown by the co-movement of the time series of the AUC and the volatility during crisis periods in Fig. 1 and in the case study around the Covid-19 crisis in Fig. 2, the co-movement between volatility and AUC is particularly strong during crisis events suggesting a potential use of this tool as specifically a crisis indicator. A quantitative interpretation of the AUC’s performance as a crisis indicator would be specific to this market and require recalibration. However, the dips before crisis suggest that defining a threshold at the long-run average of 0.84 reconstruction accuracy could serve as a first step towards building such an indicator. The out of sample results of the AUC support this interpretation as they suggest as discussed above that the GAE reconstruction encodes useful information about the instability of the market which increases during crisis.

The novelty of our contribution lies in underlining the importance of looking at node features as well as the adjacency matrix when deriving stability indicators from financial networks. We reinforce this intuition with the results of the ablation study in Table 2 and 3 which show that the combination of edge and feature information plays an important role in allowing the GAE’s reconstruction ability to act as a good explanatory variable for volatility and thus for market instability. This combination of features is important to consider when analysing networked representations of financial markets.

We argue that by showing that homogeneity of graph connections is an important feature in market stability, this work also opens avenues for future research. With that in mind we discuss some possible extensions of our work building from strengths of our approach. Our approach is flexible and can be applied to other financial networks used in the literature to describe firm relations. For example, we may apply this approach to graphs constructed from a news corpus [23, 24], supply chains [25], knowledge graphs [26] or inter-banking lending markets [27]. These other types of networks capture qualitatively different connections beyond correlations and we aim to use them to extend our research. This would allow us to go beyond the statistical correlation between returns and learn informative features derived from other types of relations, which in turn can be used to refine the volatility forecasting.

Improving volatility forecasting methods with a GAE model has a diverse array of applications in financial markets. Financial regulators need to ensure that the financial sector is robust to ‘stress-tests’ [28] which require realistic forecasting of volatility spikes such as the one proposed in the current work. The ability of our graph auto-encoder to identify volatility swings could serve as part of an early warning system in regulator’s financial policy designed to counteract shocks to the stability of the overall financial system. Meanwhile, volatility forecasting is also needed in estimating the position size, trade timing and correct pricing of derivatives which are useful tools in modern financial markets used by portfolio managers to manage risk. For example, formulas to price options such as Black-Scholes [29] need an estimate of next period volatility in order to correctly estimate the current fair value of an option. Lastly, asset allocation formulas used in portfolio management use a volatility forecasting formula to evaluate the optimal combination of stocks to put in their portfolios [30]. A stock with a higher expected volatility would then be seen as carrying more risk and our volatility forecasting could be used to bolster this dimension of financial activity. Moreover, our work also underlines the importance of shock transmission channels between firms in explaining volatility. This study using a graph autoencoder on a network of firm-to-firm relationships could be extended into other markets tied with overall financial stability, such as inter banking markets leveraging existing research in the field such as the bank and borrowing firm data used in financial policy research [31]. By helping forecast future volatility, financial regulators such as central banks can simulate how portfolios might perform under extreme market conditions, allowing them to assess and mitigate risks.

We also discuss a set of extensions that address the limitations in our solution as well as possible strategies to address them. The first tackles a limitation of the current implementation related to static embeddings: our current approach works with ‘snapshots’ of network information at subsequent time points. The embeddings the model produces is thus discontinuous through time and may not reflect the reality of fluctuations on the stock market. In future work, we would like to explore strategies to make the GAE’s representation ‘dynamic’ so that it learns and continuously updates its inner representation of the market as in DynGAE [32]. Another extension stems from the agreement between our unsupervised method using graph features and previously observed good performance of non-GAE models in finance which attempted to reconstruct only returns [11]. For example, we aim to extend the current methodology to reconstruct both the network topology and price returns simultaneously using frameworks such as the GALA architecture [33].

## Method

### Data and network construction

Our data set consists of 6 years of stock market price data for stocks in the S&P 500 at the minute-level frequency from 2015 to 2021 obtained from the EOD intra-day data API [34]. We had to drop some companies as they had no available data during the whole period leaving us with 401 companies. From these price movements which we define as $p(n,t)$ where the *nt*-th entry is the price for company *n* at *t*, we calculate a log return matrix $\mathbf{X} \in \mathbb{R}^{N \times T}$ of *N* companies over *T* time periods at frequency Δ*t*:

1 $$ r(t, n) = \text{log} (p(t, n)) - \text{log} (p(t - \Delta t, n)) $$

**Figure 4:**
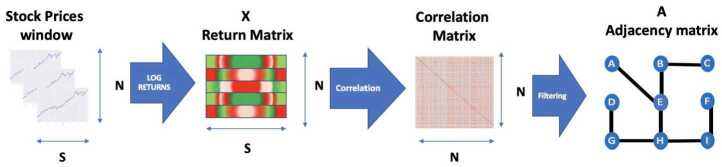
Pipeline generating networks from N returns over a window of time S

Based on this return matrix, we calculate a Pearson correlation matrix based on a rolling window of length *S* days. This correlation matrix measures for all stock return pairs the covariance normalised by the square root of the product of the variances. It represents the strength of linear relationships between all the stock pairs. Given a return matrix of *N* companies over *T*, this gives us $T-S$ correlation matrices in total. We define an adjacency matrix **A** as $\mathbf{A}_{uv} = 1$ if the (*u*, *v*) return pair has a correlation higher than 0.7 and otherwise we set $\mathbf{A}_{uv}$ to 0 following the methodology set out in [9]. The 0.7 threshold is selected to only keep strongly connected pairs and discount correlations of insufficient strengths [9, 35]. We summarise the way we construct the financial network in Fig. 4 where a series of stock prices is transformed into a return matrix.

From that return matrix, we calculate the correlation matrix as described above. Lastly, through a threshold applied to that correlation matrix, we obtain a network of connected companies. We also calculate the return volatility (RV) that is used throughout the following sections as a proxy for instability according to the formula set out in Eq. (2). It is a measure of the variance of the returns of the average price returns over Δ*t* periods. Because of the power law distribution of market volatility with orders of magnitude larger swings on high-volatility days, volatility is often measured as the natural logarithm of RV and termed log-RV.

2 $$ \text{RV}_{t}^{t+\Delta t} = \sum _{t}^{t+\Delta t}(r(t,n)^{2}) $$

### GAEs

Based on the problem setting and data description in the previous two sections, we now formally describe our methodology. Given the financial graph $\mathcal{G}$ with adjacency matrix **A** and node features **X** defined above, we propose to use the GAE [21] as the edge reconstruction model whose generalisation ability is used to approximate market instability. The GAE is an unsupervised representation learning model with an encoder and a decoder. The encoder is a function defined in Eq. (3). It describes an operation mapping the input **X** to the latent embedding **Z** via a 2-layer graph convolutional network (GCN) encoder [36].

3 $$ \mathbf{Z} = \text{GCN}(\mathbf{A},\mathbf{X}) = \tilde{\mathbf{A}}* \text{ReLU}(\tilde{\mathbf{A}}*\mathbf{X}*\mathbf{W_{0}})* \mathbf{W_{1}} $$

With the following definitions:

- $\tilde{\mathbf{A}}= \mathbf{D}^{-\frac{1}{2}}\mathbf{A}\mathbf{D}^{- \frac{1}{2}} $ is the normalised adjacency matrix of the graph. Where $\mathbf{D}=diag(d)$ is the diagonal matrix of the graph $\mathcal{G}$ where the values on the diagonal correspond to the degree of the node.
- ReLU(**x**) = max(**x**,0) is the non-linear activation function of the GCN.
- $\mathbf{W_{0}}$ and $\mathbf{W_{1}}$ are learnable weight matrices

Once the encoder generates the embedding **Z**, the decoder in equation Eq. (4) aims to reconstruct the edges present in the initial adjacency matrix.

4 $$ \forall (u,v) \in \mathcal{V}*\mathcal{V}, \hat{\mathbf{A}} = \sigma ( \mathbf{z_{u}}*\mathbf{z_{v}}) = \frac{1}{(1+e^{-\mathbf{z_{u}}*\mathbf{z_{v}}} )} $$

The embedding **Z** is of “good quality” if the reconstruction $\hat{\mathbf{A}}$ is close to the initial adjacency matrix. The GAE is trained by minimising a binary cross-entropy (BCE) loss defined in Eq. (5) on training edges $\mathbf{A}_{uv}$:

5 $$ (\mathbf{A}_{uv}, \hat{\mathbf{A}}_{uv}) = -\mathbf{A}_{uv}*log( \hat{\mathbf{A}}_{uv}) -(1-\mathbf{A}_{uv})*log(1- \hat{\mathbf{A}}_{uv}) $$

**Figure 5:**
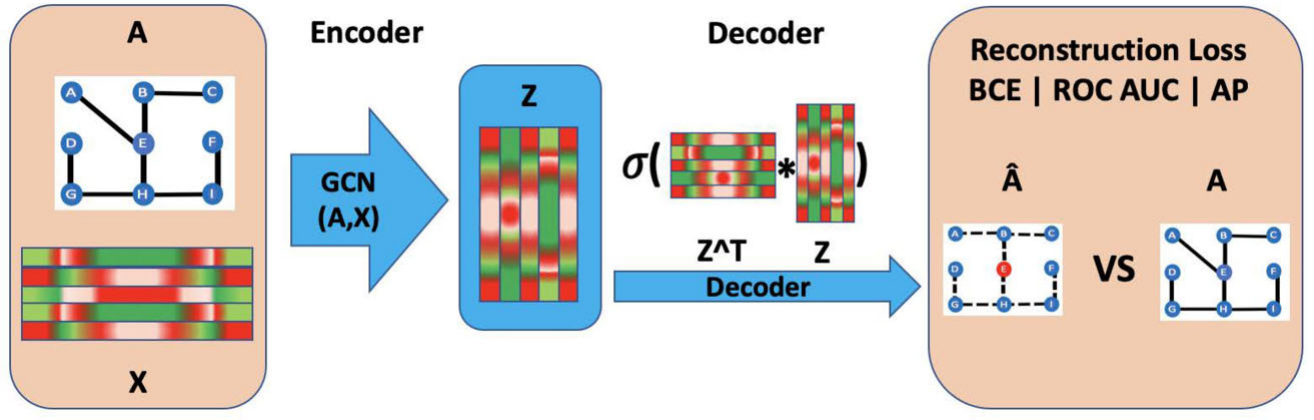
Summary of GAE operations

The end-to-end training is done using gradient descent for which we selected a standard Adam algorithm [37]. Figure 5 summarises how the GAE operates.

### Measuring market instability

We now describe how we make use of the GAE model to derive a measure of market instability. We first extract the returns data **X** from a window of length $S = 20$ days. From those returns, we generate the corresponding market network $\mathcal{G}_{t}$ with *t* denoting the last day in the time window using the methodology described in Sect. 5.1 We selected a span of 20 days as this represents one month of trading data, which is long enough to capture relations between companies without too much noise from unstable correlations in shorter windows [9]. Shifting the window by one day, we generate the next day market graph $\mathcal{G}_{t+1}$.

We train and validate the GAE on $\mathcal{G}_{t}$, and test its performance by applying the trained model to reconstruct edges in $\mathcal{G}_{t+1}$, the market graph of next day $t+1$.Given the high average in sample reconstruction accuracy of 0.84 we observe throughout our sample, we assume the GAE is able to reconstruct the edges of a market graph in a stable period. This also means that, when applied out of sample to reconstruct the edges of the market graph $\mathcal{G}_{t+1}$, we can be confident that the reconstruction error will reveal information about the market state (homogeneous or heterogeneous) rather than about the quality of the training. Following the reasoning in Sect. 2, once a GAE is trained, it can reconstruct the adjacency matrix of an unobserved graph in proportion to how homogeneous the unobserved graph is [21]. In that light, we define homogeneity in a graph by how easy it is to reconstruct by a GAE which has been properly trained. Consequently, a low GAE testing accuracy on $\mathcal{G}_{t+1}$ can be interpreted as representing an increase in graph heterogeneity. This increased heterogeneity measures how different the edge formation mechanism in $\mathcal{G}_{t+1}$ is different from $\mathcal{G}_{t}$. The high in-sample reconstruction accuracy of our GAE allows us to be confident in its’ ability to reconstruct homogeneous edge-connection patterns. It also allows us to interpret collapses in reconstruction performance in the out of sample setting as good indicators of market heterogeneity and upcoming volatility spikes. Previous results in the literature, such as [13] suggest that the change in market network homogeneity, measured by edit distance of edges between two successive time periods, granger-causes increases in market volatility. Following that approach, we interpret the difference in edge formation mechanism between $\mathcal{G}_{t}$ and $\mathcal{G}_{t+1}$ as resulting from shocks to investor opinion which push the market towards a more volatile state [11, 12]. Indeed, changes in the financial connectivity of stockmarkets is interpreted as revealing of the speed at which various market agents process new information about firms, [38] for instance, describes the instability revealing effect of modifications of low-frequency (ie daily or longer) financial connectivity such as the 21-day correlation windows we use to build our representation of the market. The link between market instability and changes in financial connectivity patterns has also been raised by the seminal work of [3] who argues that it is the breakdown of hierarchies in correlation networks (ie changes in the network structure) is a good pre-spike indicator. These shocks also form the basis of market instability, and thus we expect this change in network homogeneity between day *t* and day $t+1$ to correlate with higher volatility on day $t+2$. The reconstruction accuracy measure which we use in our paper to signify the next day edge reconstruction performance in our binary edge-reconstruction task for any given test graph $\mathcal{G}_{t+1}$ is area under receiver operating characteristic curve ($\mathbf{S}_{t+1}$).

**Figure 6:**
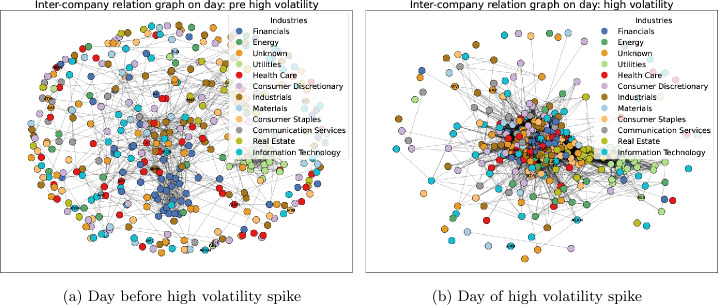
Network homogeneity before and during high volatility period

Figure 6 describes two consecutive graphs in our sample and Table 4 describes graph statistics of the two respective graphs. The left-hand image corresponds to the 21st of august 2015 a day of low volatility and the network displays high edge homophily meaning the edges connect nodes from the same industry 63% of the time, low edge density, low average clustering and multiple disconnected components. Those are all traits of regular low-volatility environments described in Sect. 2. Conversely, the right-hand side graph is the graph of the next trading date, the 24th of august, a day with higher volatility. The high-volatility date is associated with a market network with lower edge homophily of 18% (meaning edges connect firms in the same industry only 18% of the time), 20 times higher edge density, 3 times higher average clustering coefficient and 4 times fewer disconnected components compared to the previous low volatility date. This is also observable by comparing the two graphs visually. The low volatility date on the left displays a few clusters of blue (financials) and light green (utilities) industry-specific stocks with most stocks forming smaller groupings. Meanwhile the right hand side graph has most of it’s nodes connected tightly in the center in one large connected component. These differences illustrate the difference between a high-volatility day and a low volatility day in our sample. This confirms the intuition in [9, 39] that high volatility is associated with changes in the network property and edge formation mechanisms of the market. Stocks in high-volatility markets tend to become more correlated even if the increased correlation does not correspond to any form of economic interdependencies such as supply chains between industries. As such a reconstruction method such as the GAE which takes as one of its’ inputs the edge information and learns how the edge and node-level information interacts can help in detecting market

**Table 4:** Network statistic changes

Volatility	Edge homophily	Edge density	Average clustering coefficient	Connected components
Pre-volatility Spike	63.9%	0.0152	0.292	137
Volatility Spike	18.26%	0.203	0.673	31

**Figure 7:**
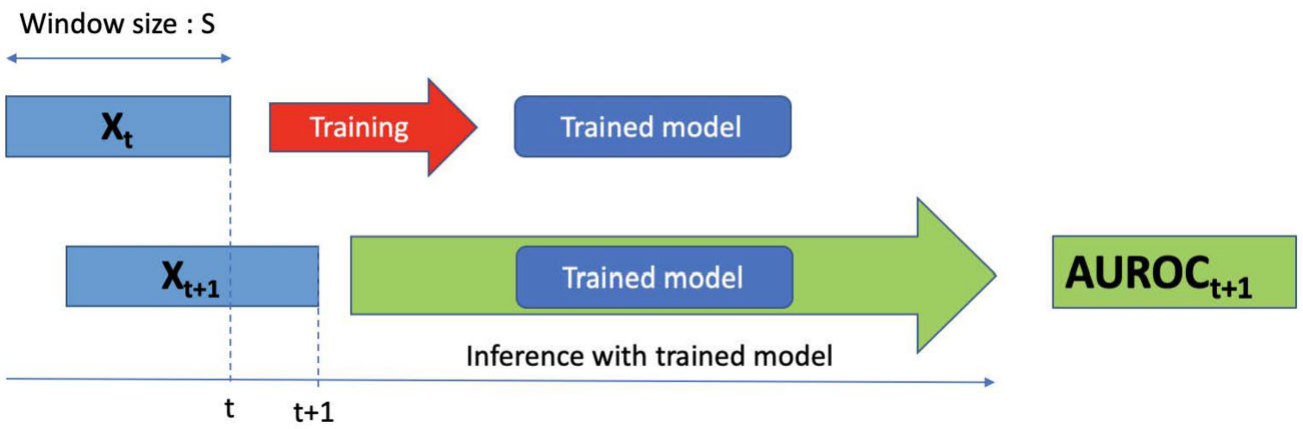
Process of generating $\mathbf{S}_{t+1}$ from model trained at *t*

As such, we use the training of the GAE only as a synthetic objective to produce $\mathbf{S}_{t+1}$ which serves only as an input variable in the downstream volatility forecasting model. The process is summarised in Fig. 7, where the shift between data at time point *t* and $t+1$ is shown as a shift of the two time series $\mathbf{X}_{t}$ and $\mathbf{X}_{t+1}$, with the former being used to train the GAE and the latter for testing.

### Volatility forecasting models

To assess the usefulness of the proposed measure, we first look at correlations between $\mathbf{S}_{t+1}$ (the next day GAE reconstruction) and $\text{RV}_{t+1}^{t+2}$ (the following day’s return volatility). This comparison is done by observing the variation of the time series of $\mathbf{S}_{t+1}$ and market volatility in Fig. 1, observing the KDE plots and the spearman’s rank correlation of $\mathbf{S}_{t+1}$ and $\text{RV}_{t+1}^{t+2}$ in Fig. 3. We chose the spearman’s rank correlation as it is less vulnerable to outliers. High correlation between $\mathbf{S}_{t+1}$ and $\text{RV}_{t+1}^{t+2}$ would indicate that changes in $\mathcal{G}_{t+1}$’s homogeneity compared with $\mathcal{G}_{t}$ are in sync with market instability.

As a second step, we test the usefulness of $\mathbf{S}_{t+1}$ for the forecasting of $\text{log}(\text{RV})$ at the following ${t+2}$ time step ($\text{log}(\text{RV}_{t}^{t+2})$). We perform this forecasting with the HAR model for log-RV -defined in (6) below- forecasting by [40]. The HAR is a strong and industry recognised benchmark model for volatility forecasting [11]. It uses auto-regressive components of lower frequency past volatility to predict future volatility. Lower frequency volatility measurements are weekly and monthly volatility. This model learns a parameter $\beta _{n}$ for each previous volatility measure from the past time periods. We look at an 1-hour frequency of forecasting as it is a frequency of interest for industry practitioners [1, 5, 11]. We calculate the $R^{2}$ of this estimation for three regressions which serve as baseline models. These are a linear regression model, a XGBoost regression tree and a multi layer perceptron (MLP) that use averaged lagged volatility as an explanatory variable as described in (6). We select hyper parameters for these models by random search and cross-validate them using a 3-fold cross-validation.

6 $$ HAR(\text{log}(\text{RV}_{t}^{t+1})) = \beta _{1}*\text{log}(\text{RV}_{t}^{t+1})+ \beta _{2}*\text{log}(\text{RV}_{t-6}^{t+1}) $$

Using the result in Eq. (6), we use the HAR-RV model for our out-of-sample volatility forecasting task to calculate the 2 hour ahead volatility $\text{log}(\text{RV}_{t+1}^{t+2})$ as a function of the autoregressive model applied on all the previous time steps’ volatility information.

7 $$ \text{log}(\text{RV}_{t+1}^{t+2}) = HAR(\text{log}(\text{RV}_{t}^{t+1})) \newline $$

To assess the usefulness of our GAE-based indicator, we run these same regressions again under an ‘improved’ setting as described in Eq. (8), by adding the $\mathbf{S}_{t+1}$ feature as an additional explanatory variable on top of averaged lagged volatility captured by the traditional HAR-RV. We compare the $R^{2}$ of the baseline models with the $R^{2}$ of the ‘improved’ models. An increase in the $R^{2}$ of the forecasting model that uses $\mathbf{S}_{t+1}$, the GAE-based signal, would suggest that the GAE encodes useful information for out-of-sample volatility forecasting.

8 $$ \text{log}(\text{RV}_{t+1}^{t+2}) \sim HAR(\text{log}(\text{RV}_{t}^{t+1})) + \beta _{3}*\mathbf{S}_{t+1} $$

To test the hypothesis that $\mathbf{S}_{t+1}$ encodes useful information, we perform a one sided t-test of the difference between the $R^{2}$ of the regular and ‘improved’ approaches by testing whether the $R^{2}$ of the proposed method is higher than the regular methods’ $R^{2}$. The $H_{0}$ null hypothesis is that the $R^{2}$ with $\mathbf{S}_{t+1}$ is not significantly larger than the one without while the $H_{1}$ is that there is a statistically significant increase in $R^{2}$ when using the $\mathbf{S}_{t+1}$ as an independent variable.

We also provide an ablation study to verify the usefulness of the GAE reconstruction accuracy in the cases where the GAE learns the node and edge features of a market graph $\mathcal{G}$ from which either the edge information $\mathcal{E}$ or the node feature matrix **X** have been replaced with uninformative values. In Table 2, we replace the feature matrix with the identity matrix when training and testing the GAE model that generates the reconstruction ROC $\mathbf{S}_{t+1}$. In Table 3, we replace the adjacency matrix with a rewired adjacency matrix - thus maintaining the initial number of edges but information represented by their connections - when training and testing the GAE model that generates the reconstruction ROC $\mathbf{S}_{t+1}$.

## Data Availability

The dataset for this research is commercially available from the EOD historical intraday API [34] available at: https://eodhistoricaldata.com/financial-apis/intra-day-historical-data-api/.

## Abbreviations

GAE: Graph auto-encoder
GCN: Graph convolutional network
S&P: Standard and Poor’s
VIX: Chicago Board Options Exchange Volatility Index
KDE: Kernel density estimation
RV: Return volatility
GARCH: Generalized autoregressive conditional heteroskedasticity
HEAVY: High-frequency-based volatility
HAR: Heterogeneous autoregressive model
ROC: Receiver operating characteristic
